# *Vismia guianensis* Improves Survival of *Tenebrio molitor* and Mice During Lethal Infection with *Candida albicans*

**DOI:** 10.3390/antibiotics14010072

**Published:** 2025-01-11

**Authors:** Arthur André Castro Costa, Elizangela Pestana Motta, Aluísio Silva Oliveira, Pamela Gomes Santos, Josivan Regis Farias, Danielle Cristine Gomes Franco, Mayara Cristina Pinto Silva, Nicolle Teixeira Barbosa, Simone Batista Muniz, Luís Douglas Miranda. Silva, Lucilene Amorim Silva, Claudia Quintino Rocha, Flavia Raquel Fernandes Nascimento, Rosane Nassar Meireles Guerra

**Affiliations:** 1 Programa de Pós-Graduação em Ciências da Saúde, Universidade Federal do Maranhão, Ensino Integrado, Bloco 3, Av. dos Portugueses, 1966, São Luís 65080-805, Maranhão, Brazil; arthur.andre@discente.ufma.br (A.A.C.C.); josivan.regis@discente.ufma.br (J.R.F.); danielle.franco@discente.ufma.br (D.C.G.F.); simone.batista@discente.ufma.br (S.B.M.);; 2 Laboratory of Immunoparasitology and Pathology, Universidade Federal do Maranhão, Av. dos Portugueses, 1966, São Luís 65080-805, Maranhão, Brazil; 3 Laboratory of Immunophysiology, Universidade Federal do Maranhão, Ensino Integrado, Bloco 1, Sala 1A, Av. dos Portugueses, 1966, São Luís 65080-805, Maranhão, Brazil; mayara.silva@ufma.br; 4 Laboratory of Chemistry of Natural Products, Centro de Ciências Exatas e Tecnológicas, Universidade Federal do Maranhão, Av. dos Portugueses, 1966, São Luís 65080-805, Maranhão, Brazil

**Keywords:** antifungal, macrophage, neutrophil, sepsis, TNFα, *Tenebrio molitor*, *Vismia guianensis*

## Abstract

**Background/Objectives**: *Vismia guianensis* is a vegetal species popularly used to treat fungal infections. This study evaluated the anti-*Candida* effect of *V. guianensis* extract after *C. albicans* lethal infection in *Tenebrio molitor* larvae and mice. **Methods and Results**: The chemical profile analysis of a hydroethanolic extract of the leaves of *V. guianensis* (EHVG) identified 14 compounds. Two sets of experiments used *T. molitor* larvae. To evaluate toxicity, the uninfected larvae were treated with EHVG or anthraquinone. We considered the following groups: the controls received PBS; ANFO B received amphotericin B (600 mg/mL); EHVG received the extract; and ANTQ received anthraquinone. The extract and anthraquinone resulted in low-level toxicity in the *T. molitor* larvae. Another set of experiments evaluated the EHVG effect during lethal infection with *Candida albicans*. The *T. molitor* larvae were treated intracelomically (ic/10 μL). Treatment with EHVG efficiently improved the survival of the larvae after lethal infection (60%), probably due to the reduction in CFUs. In the mice, the antifungal effect of EHVG was determined in three groups of immunosuppressed Swiss mice (cyclophosphamide, 50 mg/kg/ip) infected with *C. albicans* (1 × 10^7^ CFU/ip). The control animals were infected and untreated; the ANFO B animals were infected and treated with amphotericin B (600 µg/kg/ip); and the EHVG animals were infected and treated with the extract (5 mg/kg/orally). A SHAM group (uninfected and untreated) was also included. Survival was assessed for 5 days. The extract increased the mice’s survival (60%) and life expectancy, reducing the CFU counts in the peritoneum and blood. EHVG also increased the number of blood neutrophils and peritoneal macrophages. These systemic activities are likely associated with the presence of flavonoids in the extract. **Conclusions**: The beneficial effects of EHVG in lethal sepsis are related to an antifungal effect, with the number of CFUs decreasing in the larvae and the mice. In addition, EHVG showed immunological activity in the mice, considering immune cell distribution and cytokine production.

## 1. Introduction

The clinical manifestations caused by *C. albicans* infection are considered a global public health problem as they can result in clinical conditions ranging from mild to severe, such as cutaneous candidiasis, oral candidiasis, vaginal candidiasis, and sepsis [1]. It is important to note that *Candida albicans* is a commensal and opportunistic fungus that can present pathogenic behaviors as a result of changes in the natural microbiota and the immune system [1,2,3].

For the present study, we investigated the anti-*Candida albicans* properties of *Vismia guianensis* (Aubl.). Chosy a plant species popularly known as fever tree, gum seal, *pau-de-lacre*, or *lacre* [4]. *V. guianensis* is a shrub belonging to the Clusiaceae family, the subfamily Hypericoideae, the tribe Vismieae, and the genus *Vismia*. This plant species is frequently found in several geographic regions in Brazil [5].

The populations in these areas have long used *V. guianensis* to treat wounds and skin fungal infections, and furthermore as a laxative, antipyretic, and antirheumatic [6,7]. Some pharmacologically active constituents identified in *V. guianensis* extracts include anthraquinones, Vismione D, kaempferol, and quercetin, which are associated with effects on macrophage activation [8], as well as antioxidant, antibacterial, and antifungal activities [9,10,11]. According to Oliveira [10], *V. guianensis* can reduce the fungal load in the spleen of male Balb/c mice infected with *Sporothrix schenckii*.

Our group has recently shown that EHVG inhibited or reduced the essential virulence factors of *Candida albicans*, including adhesion, biofilm formation, and the production of exoenzymes. This activity was related to Vismione (among other chemical constituents), as indicated by its high affinity for some *C. albicans* molecules [11]. *Tenebrio molitor* larvae are an interesting alternative model for evaluating the toxicity of vegetal extracts and infection caused by *Candida albicans*. This invertebrate animal can be easily housed and allows for the evaluation of several doses of the one tested extract using groups with high numbers of animals.

Based on these considerations, the present study evaluated the anti-*Candida* activity of EHVG during lethal infection in mealworm beetle *Tenebrio molitor* larvae and Swiss mice, considering that in vivo data are still scarce.

## 2. Results

### 2.1. Chemical Profile of EHVG

**Figure 1:**
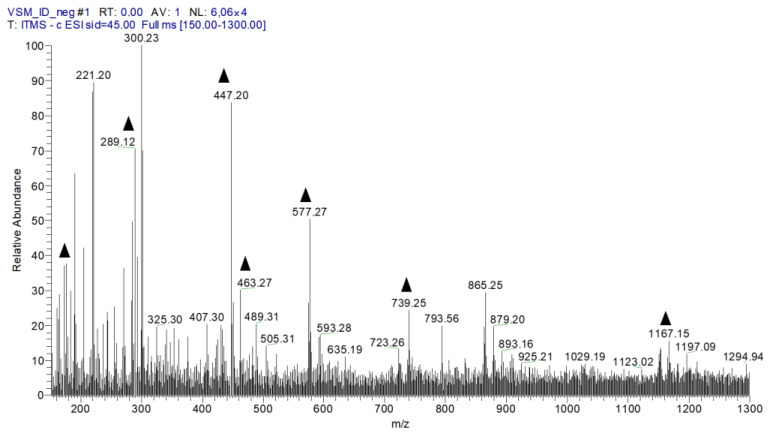
The first-order spectrum of direct flow injection analysis (FIA-ESI-IT-MS) was obtained in the negative mode for EHVG. (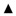) Fragments of chemical compounds identified.

Figure 1 shows the peaks of fourteen compounds found after the chemical analysis of EHVG, and Table 1 lists the names of the identified compounds found in EHVG. It is possible to observe the presence of anthraquinones and Vismione D—markers of the genus *Vismia*. The same data have previously been published by our group [11].

### 2.2. EHVG and Anthraquinone Showed Low-level Toxicity to T. molitor Larvae

Table 2 details the toxicity of EHVG and anthraquinone tested on uninfected larvae. Both the treatments presented no toxicity for doses between 1 and 50 mg/kg of body weight, as all the animals remained alive. The treatment with EHVG with a dose of 100 mg/kg ensured the survival of 60% of the larvae, while a dose of 500 mg/kg showed the highest toxicity, as only 47% of the animals survived. We did not test high doses of anthraquinone between 100 and 500 mg/kg, considering the concentration of this isolated compound. The compound’s lethality is considered by measuring melanization, activity, and the occurrence of death.

**Figure 2:**
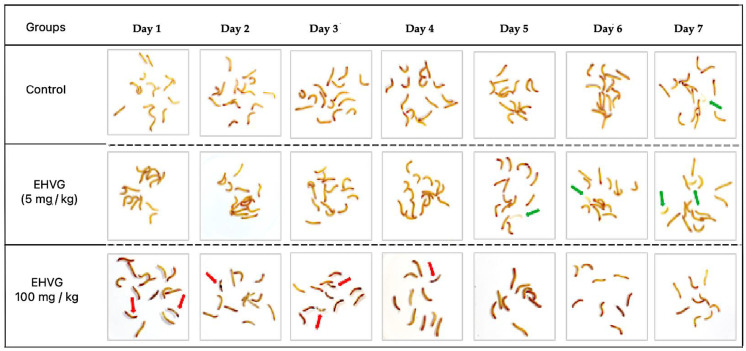
A visual representation of the toxicity of the hydroethanolic extract of *Vismia guianensis* (EHVG) in the *Tenebrio molitor* larvae. The green arrows indicate a chrysalid or chrysalid formation, and the red arrows indicate the dead larvae (n = 15/group).

Figure 2 schematically represents the toxicity of two EHVG doses (5 and 100 mg/kg) compared with that of the untreated control group.

### 2.3. EHVG and Anthraquinone Reduced the Lethality of C. albicans Infection in T. molitor Larvae

**Figure 3:**
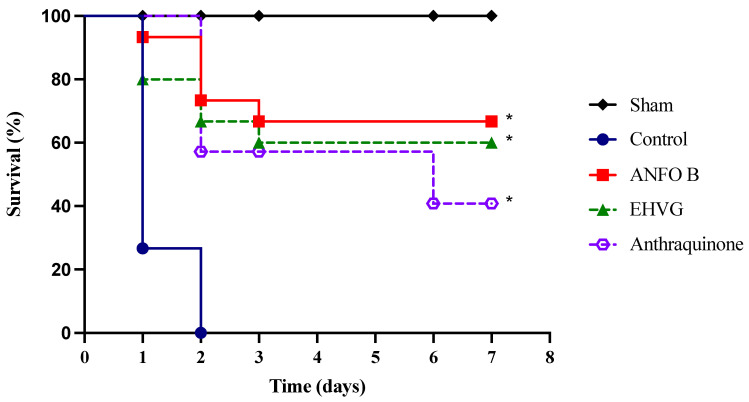
The treatment with *V. guianensis* hydroethanolic extract (EHVG) and anthraquinone increased the survival rate of the *T. molitor* larvae lethally infected with *C. albicans*. The animals were infected with 1 × 10^7^ CFU/mL (10 μL) via an intracelomic route (ic) and treated using the same route with 10 μL of EHVG (5 mg/kg, 10 μL) or anthraquinone (5 mg/kg). The EHVG-treated groups were compared to those larvae infected and treated with amphotericin B (ANFO B; 0.6 mg/kg, 10 μL) and with a control group that received PBS (10 μL). The data expressed as percentages were obtained from the Log-Rank (Mantel–Cox) test and a Kaplan–Meier curve considering 15 animals/group. (*) *p* < 0.05 in comparison to the control.

The treatment with EHVG or anthraquinone increased the survival rate of the animals lethally infected with *C. albicans*. EHVG (hydromodule 1:10) was more effective, as it ensured the survival of 60% of the larvae, while the anthraquinone treatment resulted in 41% survival. EHVG showed survival rates similar to those detected in the ANFO B group (66%). All the larvae in the infected control group died within 48 h of infection (Figure 3).

### 2.4. EHVG Inhibited C. albicans Infection in T. molitor

**Figure 4:**
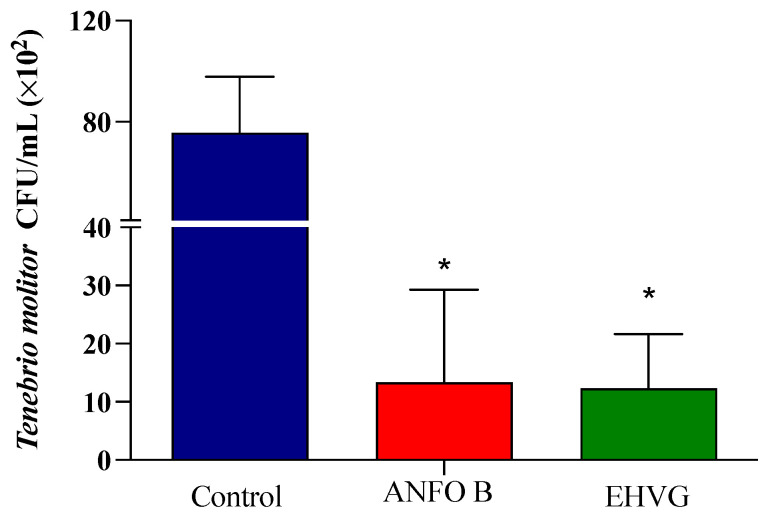
The hydroethanolic extract of *V. guianensis* leaves (EHVG) reduced the number of *C. albicans* colonies in *T. molitor*. The animals were infected with sub-lethal concentrations of *C. albicans* (5 × 10^4^ CFU/mL, ic, 10 μL), and the CFUs were determined 3 days later. The larvae were treated concomitantly (via ic route, 10 μL) with EHVG (5 mg/kg) or amphotericin B (0.6 mg/kg, 10 μL), and then compared with the control group (receiving PBS, 10 μL). The mean ± standard deviation data correspond to 15 animals/group. (*) *p* < 0.05 in comparison to the control group.

The treatment with EHVG efficiently reduced the number of CFUs in the animals lethally infected with *C. albicans* when compared to that of the control group (*p* = 0.0103). The results were similar to those obtained for amphotericin B (*p* = 0.9297; Figure 4).

### 2.5. Effects of EHGV in Mice

#### EHVG Increased the Survival and Life Expectancies in Mice Lethally Infected with *C. albicans*

**Figure 5:**
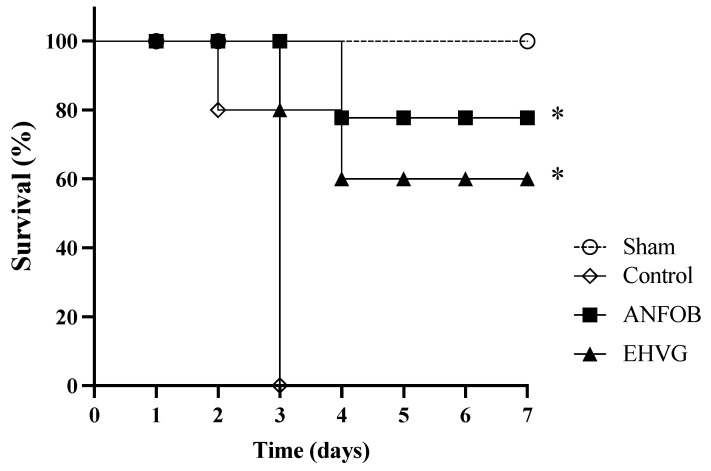
The treatment with EHVG improved the survival rate of the mice infected with *C. albicans*. The animals were immunosuppressed with cyclophosphamide (50 mg/kg, ip), infected 48 h later with *C. albicans* (1 × 10^7^ CFU, ip, 200 μL), and distributed into groups according to treatment (200 μL): EHVG, oral treatment with EHVG (5 mg/kg, 200 μL); ANFO B, treatment with amphotericin B (600 μg/kg/100 μL); and control, receiving PBS. A SHAM group without infection and treatment was also included. The data are expressed as mean ± standard deviation considering 5 animals/group. (*) *p* < 0.05 compared to PBS.

The treatment with EHVG improved the survival rate of the mice lethally infected with *C. albicans* by 60% (Figure 5). Therapy with amphotericin B also increased the survival rate after infection (78%) more, reflecting the animals’ life expectancy (Table 3) compared to that of the untreated control group, in which all the animals died three days after infection.

The EHVG and ANFO B groups had similar life expectancies, with median survival times (MSTs) corresponding to 4.4 and 4.8 days, respectively. The EHVG group’s increase in lifespan (ILS) was 91%. In all the evaluations, the results obtained for the EHVG and ANFO B groups were better than those of the control group (Table 3).

### 2.6. Treatment with EHGV Reduced the Number of CFUs in the Peritoneum and Blood

**Figure 6:**
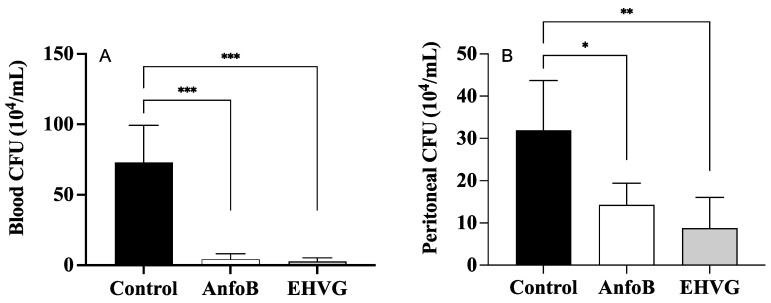
EHVG efficiently reduced the number of CFUs in the blood (**A**) and the peritoneum (**B**). The animals were immunosuppressed with cyclophosphamide (50 mg/kg, ip, 200 μL) 48 h before *C. albicans* infection (1 × 10^7^ CFU, ip, 200 μL) and divided into groups according to the treatments. EHVG, the animals were treated orally (immediately after infection) with hydroalcoholic extract of *V. guianensis* (EHGV; 5 mg/kg, 200 μL); ANFO B, the animals were treated subcutaneously with amphotericin B (600 μg/kg, 100 μL); control, the animals received PBS; SHAM, there was no infection and no treatment. The data are expressed as mean ± standard deviation (n = 5 animals/group). (*) *p* < 0.05; (**) *p* < 0.01; and (***) *p* < 0.001 when compared to the control group.

The mice treated with EHVG showed a reduced number of CFUs in the blood (Figure 6A) and the peritoneum (Figure 6B), similar to the results observed in the ANFO B group. No values were detected for the SHAM group; therefore, this information is not included in the figures.

### 2.7. Systemic Effects of EHVG on Infected Mice

**Figure 7:**
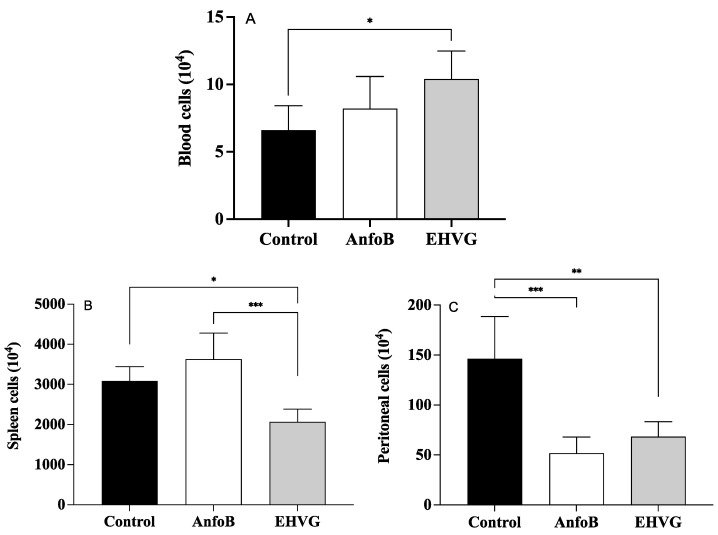
Different effects of EHVG on blood (**A**), splenic (**B**), and peritoneal cells (**C**) from infected mice. Animals were immunosuppressed with cyclophosphamide (50 mg/kg, ip, 200 μL) 48 h before *C. albicans* infection (1 × 10^7^ CFU, ip, 200 μL). Animals were distributed into following groups according to treatment that occurred immediately after infection: EHVG, received extract by oral route (5 mg/kg, 200 μL); ANFO B: received amphotericin B (600 μg/kg, 100 μL) subcutaneously; control group, received sterile PBS; and SHAM, no infection and no treatment. Cells were counted under ordinary light optical microscope (X40). Data represent mean ± standard deviation (n = 5 animals/group). (*) *p* < 0.05; (**) *p* < 0.01; and (***) *p* < 0.001 when comparing EHVG with other groups. (**) *p* < 0.01 comparing Anfo B and control group.

Treatment with the extract reduced the number of splenocytes (Figure 7A) and peritoneal cells (Figure 7B). This reduction was more pronounced than that in the control group’s spleens (Figure 7A) and peritoneum (Figure 7B). However, the treatment with EHVG increased the number of blood leukocytes (Figure 7C). Similar results were observed in the ANFO B group, except for the blood leukocyte counts, as in this group, the results were identical to those for the control group.

### 2.8. EHVG Increased the Numbers of Blood Neutrophils and Peritoneal Macrophages

**Figure 8:**
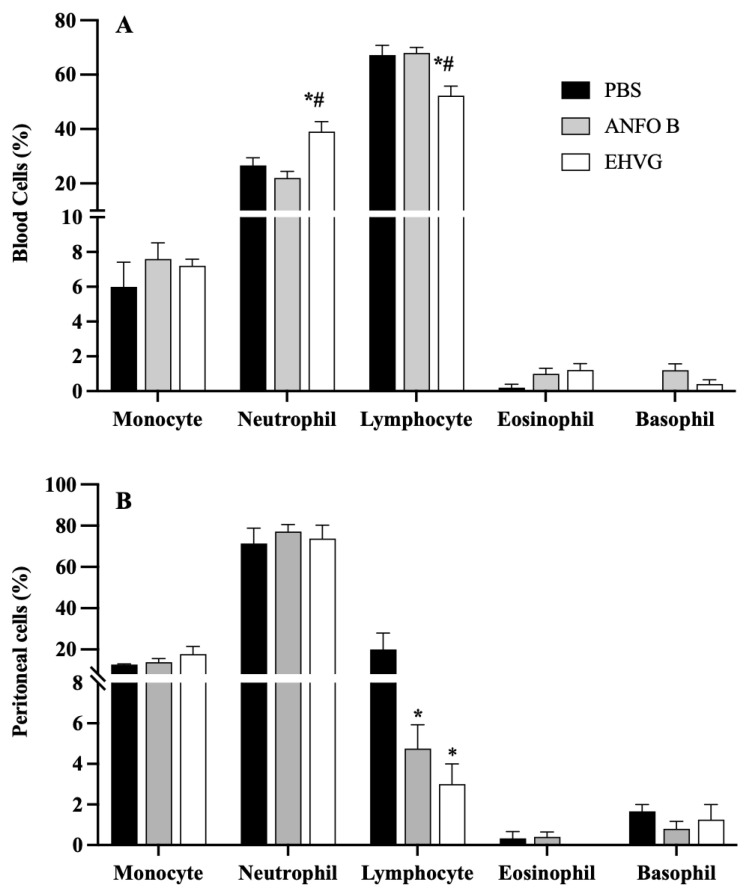
Cell populations in blood (**A**) and peritoneal fluid (**B**) from animals immunosuppressed with cyclophosphamide (50 mg/kg, ip, 200 μL) 48 h before *C. albicans* infection (1 × 10^7^, ip, 200 μL). EHVG, animals infected and treated (immediately after infection) orally with hydroalcoholic extract of *V. guianensis* (5 mg/kg, 200 μL); ANFO B, animals infected and treated subcutaneously with amphotericin B (600 μg/kg, 100 μL); PBS, infected animals that received PBS (control). Data are expressed as mean ± standard deviation (n = 5 animals/group). (*) *p* < 0.05 when compared to control, and (#) *p* < 0.05 when compared to the ANFO B group.

The differential blood cell count showed that the EHVG treatment increased the number of neutrophils and reduced the number of lymphocytes when compared to those of the ANFO B group (Figure 8A). In the peritoneum, the treatment with EHVG increased the number of peritoneal macrophages, but did not affect the neutrophil, lymphocyte, eosinophil, or basophil counts (Figure 8B).

### 2.9. Treatment with EHVG Reduced TNF-α Levels and Increased IL-10 Levels During Infection

**Figure 9:**
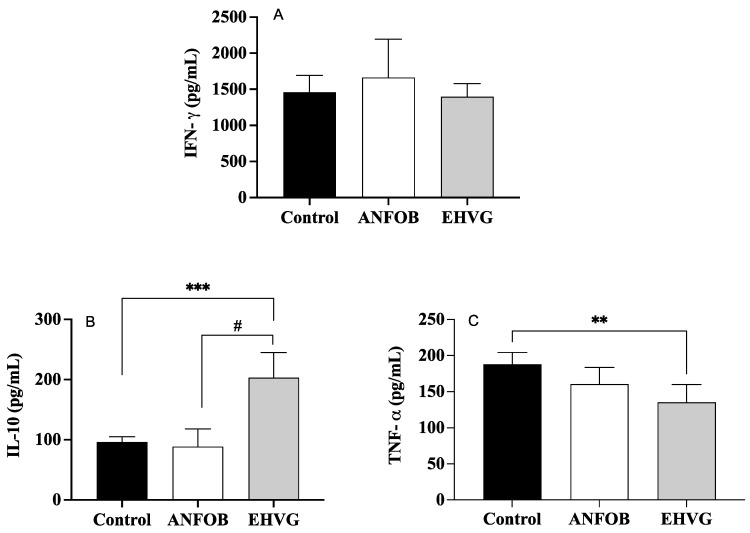
Treatment with EHVG modulates cytokine levels: IFN-γ (**A**), IL-10 (**B**), and TNF-α (**C**). Animals were immunosuppressed with cyclophosphamide (50 mg/kg, ip, 200 μL) and lethally infected 48 h later with *C. albicans* (1 × 10^7^ CFU, ip, 200 μL). Animals were divided and treated immediately after infection according to the following groups: EHVG, treated orally with hydroalcoholic extract of *Vismia guianensis* (5 mg/kg, 200 μL); ANFOB, treated subcutaneously with amphotericin B (600 μg/kg, 100 μL); PBS, infected animals that received sterile PBS (control). Data are expressed as mean ± standard deviation. (**) *p* < 0.01, and (***) *p* < 0.001 compared to control, and (#) *p* < 0.05 compared to ANFOB group.

Figure 9 shows no effect of EHVG on the IFN-γ levels (Figure 9A), which is in contrast with the increased levels of IL-10 (Figure 9B). It is also possible to observe a reduced level of TNF-α (Figure 9C) in the group treated with EHVG. The ANFO B group exhibited results similar to those of the control group for all the cytokines evaluated.

## 3. Discussion

The prevalence of *Candida* sp. infection and its increased resistance to conventional antifungals have led to a critical shortage of effective treatments for candidiasis [12,13]. Our previous results revealed that EHVG effectively inhibited *Candida* sp. in vitro, but data related to the antifungal activity of *Vismia guianensis* in vivo are still scarce. In light of this, we evaluated the chemical profile and the efficacy of EHVG in controlling the lethal infection induced by *Candida albicans* in *Tenebrio molitor* larvae and mice.

The direct flow injection analysis of EHVG confirmed the presence of phenolic compounds, including anthraquinones, catechins, epicatechins, kaempferol, Vismione, and flavonoids, as previously described by Motta et al. [11], as our work was performed with the same batch of extract.

The EHVG microbiological control effect was determined for the growth of bacteria and fungi, and no group of microorganisms was detected in all the tested samples, confirming the microbial control quality of this extract (Supplementary Materials). The microbiological quality of any product—natural or synthetic—is undoubtedly of the utmost importance in determining its use [14].

Our results demonstrated that EHVG has an anti-*Candida* effect in vivo, as the treatment with the extract increased the lifespan of the *T. molitor* larvae and the mice, while significantly reducing the CFU counts. The antifungal amphotericin B was chosen as the positive control, considering that resistance to this antifungal is rare [15]. In addition, amphotericin B targets ergosterol in the fungal membrane, impairing its integrity in a similar manner that we believe EHVG acts, according to our in-silico data published previously elsewhere [11]. This membrane instability results in fungal cell death due to a severe disturbance in ionic imbalance with loss of K+ in the extracellular medium [13,16,17].

Our previous results [11] indicated that treatment in vitro with EHVG efficiently controlled several *C. albicans* virulence factors, such as adhesion, biofilm formation, and the production of exoenzymes. In addition, the molecular docking analysis results showed that the compounds in the extract have a high binding affinity to the *C. albicans* CaCYP51 enzyme. The enzyme CaCYP51 converts lanosterol to ergosterol [18], a target of therapeutic antifungal treatments such as amphotericin B [13]. Thus, the data described in the present work associated with the previous results obtained in vitro and in silico suggest that treatment with EHVG (in *T. molitor* and mice) has a direct action on *C. albicans*, inhibiting ergosterol synthesis. In addition, the extract can also activate an immune response, resulting in an increased lifespan.

Despite the potent antifungal effect against *C. albicans*, amphotericin B is limited by its nephrotoxicity, even for liposome formulas [16,17]. Conversely, EHVG and anthraquinone showed low-level toxicity in vitro or in vivo at concentrations between 1 and 50 mg/kg, as all the larvae remained alive (less than 10% of deaths), which was comparable to the control group (Table 3); however, it should be noted that doses of EHVG greater than 100 mg/kg killed at least 60% of the larvae.

The previous results from our group using in vitro assays with RAW 264.7 cells at doses between 0.5 and 10 mg/mL [11] also indicated the low-level toxicity of the extract. This finding underscores the safety of the tested compounds and ensures its viability and dose–treatment selection, considering that 5 mg/kg can be used for further evaluation in *T. molitor* and mice, as no adverse effects were observed at this dose.

The inoculum selected for the lethal infection of *T. molitor* was able to kill all the animals in the control group within 48 h, thereby establishing a robust comparative group to evaluate the effects of EHVG. Amphotericin B had a survival percentage of 67% in the larvae.

The survival percentages in the *T. molitor* groups treated with EHVG and anthraquinone were comparable to that in the amphotericin B group, demonstrating the antifungal potential of our protocol as an alternative or complementary treatment. The increased survival rate of the *T. molitor* larvae observed in the EHVG group can be related to the reduction in CFUs observed in this group, which showed similarities to amphotericin B on days 1 and 6. This is significant in the context of our study, as *T. molitor* possesses a complex innate cellular and humoral immune system that plays a crucial role during fungal infection, as shown by the melanization process that is associated with hemolymph coagulation, the synthesis of reactive oxygen species, and the antimicrobial response [19,20]. Although we did not perform studies to establish such a correlation between the activation of immune system cells in *T. molitor*, it is reasonable to propose that EHVG also affects the hemocyte cellular response, and the humoral response includes the melanization process [21,22].

Our findings in vivo align with the previous results obtained in vitro by our group [11] and others showing the antifungal activity against *C. albicans* for extracts of *V. guianensis* leaves [6,11] and essential oils [23,24]. Furthermore, other studies using *V. guianensis* have also shown its activity against *C. albicans* and *C. glabrata* in vitro [11], indicating a promising avenue for future research and the development of new antifungals. However, as EHVG was more effective than anthraquinone, we continued our investigations only using this extract.

The mice treated with EHVG showed a reduced cell population compared to the SHAM group without infection or treatment, confirming its immunosuppressive effect, which was crucial for our study. Based on these results, we evaluated the profile of cell distributions in blood, the spleen, and peritoneal fluid. As a result, the treatment with EHVG reduced the spleen and peritoneal cell numbers while increasing the number of blood leukocytes.

The treatment with EHVG increased the IL-10 levels, the numbers of blood neutrophils and peritoneal macrophages, and reduced the TNF-a levels. Neutrophils play a fungicidal role during *C. albicans* infection due to several mechanisms, including phagocytosis, the degranulation of perforins [25], the generation of reactive oxygen species, and the ability to release NETs (neutrophil extracellular traps) [25,26]. Therefore, the increase in the neutrophil population in the EHVG group suggests that EHVG may be an additional way to control *C. albicans* infection and reduce its lethality in mice. In addition, the increased number of peritoneal macrophages may be related to the elimination of *C. albicans* in the first hours of infection, as evidenced by the reduced number of CFUs in this site, contributing to inhibiting the progression of candidemia, given that macrophages show potent antifungal activity, as described previously [27,28].

## 4. Materials and Methods

### 4.1. Botanical Identification and Preparation of Hydroethanolic Extract of Vismia guianensis (EHVG) and Its Chemical Evaluation

Leaves of *V. guianensis* were collected in São Luís, MA, at coordinates 2°28′47.1″ S 44°13′17.4″ W. Prof. Dr. Eduardo Bezerra confirmed botanical identification at the Herbarium of Maranhão (MAR), where the specimen was deposited under n° 11.078. According to Brazilian legislation Law No. 13,123/15, this study is registered in the National System for the Management of Genetic Heritage and Associated Traditional Knowledge (SisGen) under registration no. AFE7A08.

For extract preparation, the leaves collected in September 2022 were dried and crushed, and powder obtained was used for maceration in ethanol/water (7:3) for seven days, considering the hydromodule ratio 1:10 (1 g of leaves powder in 10 mL of ethanol 70%). At the end of the maceration process, solvent was removed via rotaevaporation, and the extract was lyophilized. The powder was then refrigerated until use. Ethanol was chosen due to the low-level toxicity of this solvent compared to that of others and based on the previous results obtained in our lab [11].

The chemical profile of the extract was determined using an HPLC system (Shimadzu Corp., Kyoto, Japan) equipped with a UV-VIS detector (SPA-10A) and a Luna C18 column (150 × 4.6 µm, 5 µm, 100 A) using acetic acid (2% in water) and methanol as solvents for elution at a gradient from 5% to 60% methanol over 60 min. The flow rate was 1 mL/min at room temperature, and data were collected and processed using LC Solution software (https://www.shimadzu.com/an/products/software-informatics, accessed on 10 October 2024. Shimadzu Corp., Kyoto, Japan) [29].

To analyze EHVG via FIA-ESI-IT-MS^n^, 10 mg of the crude extract was dissolved in MeOH: H_2_O (1 mL, 1:1 *v*/*v*). The extract was incubated in an ultrasound bath for 5 min, filtered (0.22 μm), and injected as aliquots (20 µL/5 ppm) into the system. Full-scan mass spectra were recorded in the range of 100–1000 m/z. Multi-stage fragmentations (ESI-MS^n^) were assessed using the collision-induced dissociation (CID) method. To identify the compounds detected in EHVG, characteristic fragmentations in the UV spectra were compared with data from the literature.

### 4.2. Candida Albicans Inoculum

This study used the standard strain of *C. albicans* (ATCC 10231) from the Immunophysiology Laboratory of the Federal University of Maranhão, São Luís, MA, Brazil. Fungal isolates were cultured in Sabouraud Dextrose Agar medium for 24 h at 35 °C. After incubation, the blastoconids were counted using an optical microscope (Nikon, Tokyo, Japan) and adjusted for sub-lethal and lethal inoculum according to a dose–response curve determined in the *Tenebrio molitor* larvae.

### 4.3. Assays with Tenebrio molitor Larvae

The *T. molitor* larvae (250 larvae), with an average weight of 100 mg, were kept at a constant temperature of 27 °C in a dark environment and with free access to food (wheat bran) throughout this study, except during experimental manipulation. The larvae belong to Biofabrica, São Luis, Ma, Brazil (https://biofabricasaoluis.com.br, accessed on 10 October 2024).

**Figure 10:**
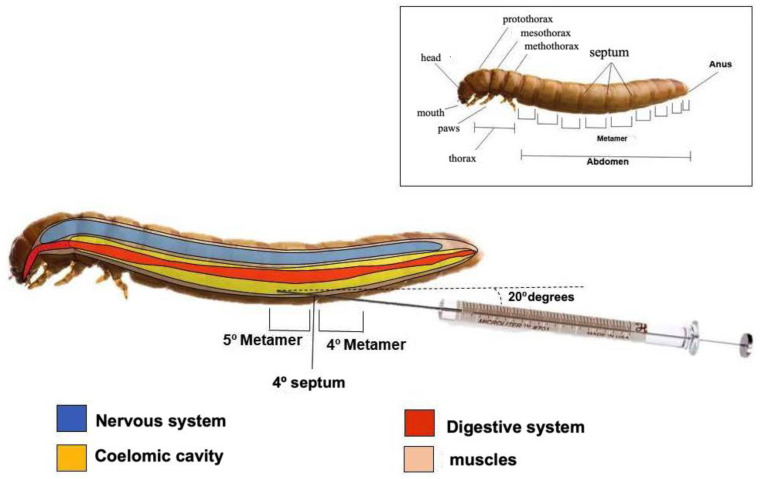
Schematic representation of *Tenebrio molitor larvae* showing site for infection and treatment between 4th and 5th metamers (^®^Biorender). To evaluate *T. molitor* lethality, the following aspects were considered: Activity—movement without stimuli (0) or the absence of movement (2). Melanization—those without melanization (0), or black larvae (2). Survival—whether they were alive (0) or dead (2).

All larvae treatments and infections were performed via intracelomic injection (10 μL) in the 4th septum (between the 4th and 5th metamers) using a Hamilton microsyringe and a fixed needle (Sigma, Sao Paulo, Brazil) in the anterior–posterior direction at an angle of 20° (Figure 10).

The larvae were weighed and divided into 11 groups (n = 15 animals/group) according to the protocol reported in Table 4, considering the different doses of EHVG and anthraquinone. After treatment, the larvae were kept in separate Petri dishes and incubated in darkness at 30 °C. Viability was assessed daily based on a change in color (melanization) and the touch response.

To evaluate toxicity, the uninfected larvae were treated with the extract or anthraquinone and compared to the untreated SHAM and the control groups that received PBS, as shown in Table 4.

To evaluate sub-lethal infection, the *T. molitor* larvae (n = 15 animals/group) were infected with *C. albicans* (5 × 10^4^ CFU/mL). In another set of experiments, the larvae received 1 × 10^7^ CFU/mL to assess their survival during lethal infection. Intracelomic treatment (10 μL) occurred just before infection for the following groups: control, received sterile PBS; ANFO B, received amphotericin B (0.6 mg/kg); EHGV, received the extract (5 mg/kg); and ANTQ, received anthraquinone (5 mg/kg). The doses of EHVG and ANTQ were determined according to their toxicity.

After treatment, the larvae were kept in separate Petri dishes and incubated in darkness at 37 °C for 7 days. The larvae’s survival was determined daily, considering their change in color and the absence of a touch response.

The number of CFUs was determined at 24 h and 3 days after infection (n = 15 animals/group for each interval). The animals were randomly selected for counts, transferred to tubes, and crushed in sterile PBS. The suspension was serially diluted at 10^−1^ to 10^−5^ concentrations and seeded on Sabouraud Dextrose Agar for 24 h at 35 °C. Figure 10 shows a schematic image regarding the site of treatment and infection, as well as the anatomy of *T. molitor* larvae.

### 4.4. C. albicans Infection of Mice

Female Swiss mice aged 8 and 10 weeks and with an average weight of 35g belonged to the Central Vivarium of the Federal University of Maranhão (UFMA). The animals had 12 h light/dark cycles and free access to food (Purina^®^ chow) and water throughout this study. The Committee on Ethics and Research in the Use of Animals (CEUA) of UFMA approved the project—protocol number 23115.037942/2018-99.

The mouse study was divided into two trials: the first aimed to evaluate survival after lethal infection, and the second aimed to assess hematological, cellular, and immunological parameters. According to treatment, the animals were subdivided into four groups in both assays (Table 5). All the experimental groups (excluding the SHAM) were immunosuppressed with cyclophosphamide (50 mg/kg, intraperitoneal route, ip; Genuxal^®^) 48 h before infection with *C. albicans* (ATCC 10231; 1 × 10^7^ CFU/mL/ip/200 μL) [30]. For assays, the animals received xylazine (20 mg/kg) and ketamine (25 mg/kg) intramuscularly 48 h after infection and treatment.

#### 4.4.1. Evaluation of Colony-Forming Units (CFUs)

Aliquots of 10 μL of whole blood (obtained by retro-orbital puncture) and peritoneal fluid were diluted (1:1000), plated on YPD agar (Sigma, St. Louis, MO, USA) and incubated at 37 °C for 24 h to determine the number of Colony-Forming Units (CFUs).

#### 4.4.2. Hematological Parameters

Smears were prepared with whole blood samples, and serum was obtained after 10 min of centrifugation (1200 rpm) at 4 °C. Blood smears were prepared with 10 μL of blood and stained using an Instant Prov kit (Newprov, Brazil) [31].

#### 4.4.3. Collection and Counting of Peritoneal and Splenic Cells

Peritoneal cells were aseptically collected in the peritoneal cavity using 5 mL of cold, sterile PBS. Spleens were collected, weighed, and crushed in sterile PBS (3 mL) to obtain cell suspensions. Peritoneal and splenic cell suspensions were stained with crystal violet (0.05% in 30% acetic acid in a 9:1 ratio) and counted in a Neubauer chamber using a light optical microscope at 400× magnification.

Cell suspensions were centrifuged in a Cytospin for differential counts and stained with an Instant-Prov kit (Newprov, Pinhais, Brazil).

#### 4.4.4. Quantification of Cytokines via Cytometric Bead Array (CBA)

Concentrations of IFN-γ, TNF-α, and IL-10 were determined in the serum samples (see Section 4.4.2) according to the manufacturer’s instructions (BD Bioscience, San Jose, CA, USA).

### 4.5. Statistical Analysis

The results were analyzed using Student’s *t*-test or analysis of variance (ANOVA), followed by a multiple comparison test (Tukey) after a normality test. Kaplan–Meier curves determined the survival data, and a Log-Rank (Mantel–Cox) test was applied to compare the curves. All analyses were performed using GraphPad Prism 10 software, and the data are expressed in graphs based on mean ± standard deviation. Differences were considered significant when *p* ≤ 0.05.

## 5. Conclusions

The anti-*Candida* effect of EHVG is probably related to several parameters acting together, similar to those factors influencing better outcomes and resolution during relevant human systemic infection. In this study, EHVG treatment increased the circulating neutrophil, peritoneal macrophage, and IL-10 levels. Simultaneously, the treatment impacted immune cell distribution and reduced the systemic CFU numbers and the TNF-α levels, thus increasing the animals’ lifespan and life expectancy. Altogether, the results demonstrated the anti-*Candida* activity of EHVG in vivo, highlighting its potential as an adjuvant treatment for fungal sepsis, particularly when administered orally. It is also reasonable to propose that EHVG may be a relevant target for the bioprospection of antifungal agents. Our findings hold promise for the future of anti-*Candida* treatments.

## Figures and Tables

**Table 1:** Compounds identified via MS^n^ in *Vismia guianensis* extract.

Nº	[M−H]^−^	MS^n^ Ions	Identified Compound
1	191	173, 111, 85	Quinic acid
2	431	269	Isovitexin
3	447	429, 357	Isoorientin
4	289		Catechin
5	447	429, 301, 269, 229	Orientin
6	431	285, 163	Kaempferol-o-rhamnoside
7	731	285, 255	Kaempferol galactoside-rhamnoside
8	1153	1001, 983, 789	Catechin tetramer
9	463	301, 283, 273, 229, 179, 121	Quercetin glycoside
10	577	425, 407, 285, 257, 213	Catechin dimer
11	1167	1015, 863, 711	A-type procyanidin trimer
12	409	273, 255	Vismione D
13	283	269, 239	Anthraquinone F
14	285		Kaempferol

**Table 2:** Toxicity of EHVG and anthraquinone in *Tenebrio molitor* larvae.

Groups ^a^	Living (No.)	Dead (No.)	Survival (%) ^b^
Sham	15	0	100
Control	15	0	100
EHVG1 ^c^	15	0	100
EHVG5	15	0	100
EHVG50	14	1	93.3
EHVG100	9	7	60 *
EHVG500	7	9	46.6 *
ANTQ1 ^d^	15	0	100
ANTQ5	15	0	100
ANTQ50	15	0	100

^a^: (ic), treatment using intracoelomic route. ^b^: Log-Rank test (Mantel–Cox); ^c^: EHVG, hydroethanolic extract from the leaves of *Vismia guianensis*; ^d^: ANTQ, anthraquinone; (*) *p* ≤ 0.05 compared to the control group.

**Table 3:** Life expectancy of animals with sepsis induced by *C. albicans*, treated with *V. guianensis* extract, and observed for survival for five days.

GROUPS ^c^	MST ^a^ (Days)	ILS ^b^ (%)
SHAM	7	100
CONTROL	1	10
ANFO B	4.8 *	96 *
EHVG	4.4 *	91 ^#^

(^a^) MST, median survival time; (^b^) ILS, increase in lifespan; (^c^) 5 animals/group; (*) *p* < 0.05 when compared to the control group; (#) *p* < 0.05 compared to the ANFO B group.

**Table 4:** Experimental groups of *Tenebrio molitor larvae* observed for acute toxicity.

GROUPS ^a^	TREATMENT (10 µL/ic ^b^)
Sham	None
Control	PBS
EHVG1 ^c^	EHGV 1 mg/kg ^d^
EHVG5	EHGV 5 mg/kg
EHVG50	EHGV 50 mg/kg
EHVG100	EHGV 100 mg/kg
EHVG500	EHGV 500 mg/kg
ANTQ1 ^e^	Anthraquinone 1 mg/kg
ANTQ5	Anthraquinone 5 mg/kg
ANTQ50	Anthraquinone 50 mg/kg

^a^: n = 15 larvae/group; ^b^: ic, intracelomic; ^c^: EHVG—hydroethanolic extract of *Vismia guianensis*; ^d^: mg/Kg of body weight; ^e^: anthraquinone.

**Table 5:** Groups for evaluation of *Candida albicans* infection of mice.

Groups	Treatment and Infection
SHAM	Without immunosuppression, infection, or treatment.
CONTROL	Immunosuppressed mice infected with *C. albicans* and treated orally with sterile PBS.
ANFO B	Immunosuppressed mice infected with *C. albicans* and treated intraperitonially with amphotericin B (0.6 mg/kg).
EHVG	Immunosuppressed mice infected with *C. albicans* and treated orally with EHVG (5 mg/kg).

## Data Availability

The data will be made available upon reasonable request.

## Supplementary Materials

The following supporting information can be downloaded at: https://www.mdpi.com/article/10.3390/antibiotics14010072/s1, Supplementary Figure S1: Supplementary data showing the safety of EHVG regarding microbiological contamination.

## Abbreviations

ACCP, American College of Chest Physicians; AMIB, Brazilian Association of Intensive Care Medicine; CEUA, Ethics Committee on the Use of Animals; EHVG, hydroethanolic extract from the leaves of *Vismia guianensis*; ic, intracelomic; ip, intraperitoneal; IL, Interleukin; IFN, Interferon; LIF, Immunophysiology Laboratory; NO, nitric oxide; PBS, phosphate-buffered saline; SCCM, Society of Critical Care Medicine; TNF-α, tumor necrosis factor alpha; CFU, Colony-Forming Unit; WHO, World Health Organization.
