# Supplementary material for: Vismia guianensis Improves Survival of Tenebrio molitor and Mice During Lethal Infection with Candida albicans
Source: Antibiotics (Basel). 2025 Jan 11;14(1):72. doi: 10.3390/antibiotics14010072 (PMC11762393; doi:10.3390/antibiotics14010072)

Supplementary Figure S1: Supplementary data showing the safety of EHVG regarding microbiological contamination; The tests were performed on Sabouraud agar (A1, A2) and Brain Heart Infusion medium (B1, B2). The extract was diluted 1:2 (A1, B1) and 1:10 (A2, B2), and the plates were incubated at 37 ºC for 48 hours. The microbiological evaluation of the *Vismia guianensis* extract (EHVG) showed no contamination by fungi or bacteria.

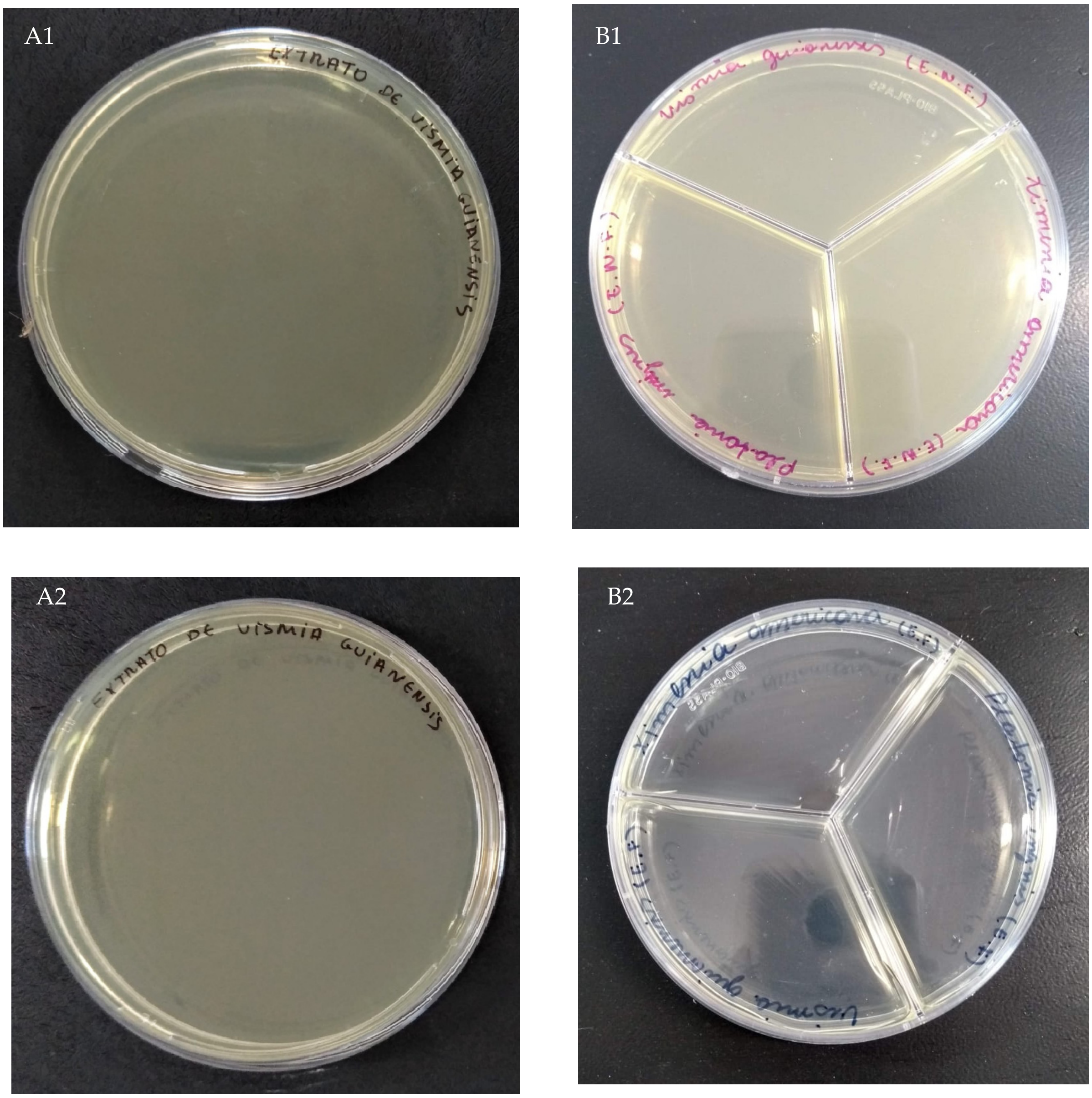

Supplement: Supplementary file 1 [file antibiotics-14-00072-s001.zip › antibiotics-3318995-supplementary.pdf]
